# From synchrotron radiation to lab source: advanced speckle-based X-ray imaging using abrasive paper

**DOI:** 10.1038/srep20476

**Published:** 2016-02-05

**Authors:** Hongchang Wang, Yogesh Kashyap, Kawal Sawhney

**Affiliations:** 1 Diamond Light Source Ltd, Harwell Science and Innovation Campus, Didcot, OX11 0DE, UK

## Abstract

X-ray phase and dark-field imaging techniques provide complementary and inaccessible information compared to conventional X-ray absorption or visible light imaging. However, such methods typically require sophisticated experimental apparatus or X-ray beams with specific properties. Recently, an X-ray speckle-based technique has shown great potential for X-ray phase and dark-field imaging using a simple experimental arrangement. However, it still suffers from either poor resolution or the time consuming process of collecting a large number of images. To overcome these limitations, in this report we demonstrate that absorption, dark-field, phase contrast, and two orthogonal differential phase contrast images can simultaneously be generated by scanning a piece of abrasive paper in only one direction. We propose a novel theoretical approach to quantitatively extract the above five images by utilising the remarkable properties of speckles. Importantly, the technique has been extended from a synchrotron light source to utilise a lab-based microfocus X-ray source and flat panel detector. Removing the need to raster the optics in two directions significantly reduces the acquisition time and absorbed dose, which can be of vital importance for many biological samples. This new imaging method could potentially provide a breakthrough for numerous practical imaging applications in biomedical research and materials science.

Ever since Röntgen’s discovery of X-rays in the late 19th century, there has been continuous development of apparatus to create, manipulate, and detect X-rays. X-ray absorption imaging provides excellent contrast between regions in the sample with dissimilar atomic numbers or density, such as bone and soft tissue. Since X-rays can penetrate deep inside objects which are opaque to visible light, X-ray absorption imaging is now ubiquitous in the modern world and is used for a wide range of everyday applications, including medical radiography, non-destructive testing, and security screening. However, for samples which have sub-structures with weak or very similar attenuation properties, such as soft tissue, X-ray absorption imaging cannot provide sufficient contrast. In the late 1930’s, Zernike developed an improved optical microscope utilising phase contrast illumination which provided substantially enhanced contrast for weakly absorbing samples. Mainly due to non-availability of high quality X-ray beams and suitable optics, it was not until 1965 that phase-contrast imaging was firstly demonstrated in the X-ray regime^1^. An alternate method to increase image contrast by improving the signal-to-noise ratio is to analyse only scattered light. Pioneers of visible light microscopy in the 17th century invented so-called dark-field techniques, where only light scattered by the sample is collected by the detector. This creates images with enhanced interfacial contrast, even at the boundary between regions with only small differences in refractive index. The main limitation of this technique is the need for a bright source to ensure sufficient scattered light reaches the detector. In the 1970s it was realized that accelerators for particle physics could provide more intense X-rays compared to conventional X-ray tubes. Throughout the 1980s and onwards, dedicated synchrotron light facilities were built to produce ever brighter X-ray beams. With improved X-ray sources, and corresponding developments in X-ray optics and detectors, X-ray dark-field imaging was developed in the late 1990s^2^. In parallel, various phase contrast X-ray imaging techniques were invented to distinguish increasingly fine details and subtle differences in sample density^3^,^4^,^5^,^6^. Complementary information provided by these different techniques has led to extraordinary achievements in biomedical imaging and materials inspection^7^,^8^,^9^,^10^,^11^,^12^,^13^,^14^,^15^. However, many such techniques are still limited by stringent requirements for the X-ray beam, in terms of spatial and temporal coherence, and monochromaticity. These techniques require complicated experimental set-ups, ultra-stability, or precision optics^16^,^17^,^18^,^19^,^20^. Although significant developments have been made to extend these techniques to practical imaging applications, many challenges still remain to be overcome.

Recently, an X-ray speckle tracking (XST) technique has been developed for two-dimensional (2D) and three-dimensional (3D) phase contrast imaging using a simple experimental arrangement^21^,^22^,^23^,^24^. However, this method suffers from poor spatial and angular sensitivity. To circumvent these limitations, 2D raster scans were proposed^25^, but this is not ideal for applications requiring fast scans and low X-ray doses. Although efforts have been made to apply XST to X-ray microfocus sources, the acquisition time is very long if a high resolution X-ray camera is used, even with the increased flux generated by a state-of-the-art liquid-jet X-ray source^26^. A recent work reported that it took nearly 30 hours to collect the entire dataset using 2D raster scans^27^. Such prolonged acquisition periods severely limit the applicability of the existing speckle-based technique for practical X-ray imaging applications.

In this report, we describe a new X-ray speckle-based approach to simultaneously collect X-ray absorption, phase contrast, and dark-field images by shining X-rays through abrasive paper. Furthermore, we have extended this approach from a synchrotron radiation source to a lab-based microfocus X-ray source by using absorption contrast speckle instead of the conventional phase contrast speckle. Consequently, both the field of view and the acquisition time have been significantly improved by using a flat panel detector with a larger pixel size. To demonstrate the capabilities of the proposed technique for biomedical research and pre-clinical applications, we present results from a *Poecilia wingei* fish and the tip of a chicken’s wing using synchrotron light and a microfocus X-ray source, respectively.

We begin by describing how the use of abrasive paper enables random speckle patterns to be generated using either synchrotron light or a lab-based X-ray microfocus source. As shown in Fig. 1, the experimental arrangements for the two types of X-ray sources are similar and relatively simple. No special optical elements are required: only a piece of abrasive paper, the sample, and an X-ray imaging detector are involved. However, it should be emphasized that the principle to produce X-ray ‘speckle’ patterns for the two cases are different. At the sample position, the typical transverse coherence length of an X-ray beam from a synchrotron source is a few tens of micrometres. When such a partially coherent beam passes through the abrasive paper with a grain size of a few micrometres, the speckle pattern caused by interference of the randomly scattered radiation can easily be observed with a high spatial resolution, X-ray detector. An important parameter to evaluate speckle quality is visibility *V*, since higher speckle visibility can significantly improve tracking accuracy when applying a cross-correlation algorithm^28^. Here, we define speckle visibility *V* as the ratio 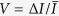, where 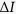 and 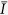 are the standard deviation and mean of the speckle pattern in the region of interest.

It is difficult to apply the speckle-based techniques with a conventional lab-based X-ray source since the effective X-ray source size is typically hundreds of micrometres and the coherence length is too short. Lab-based, microfocus X-ray generators can create a focal spot which is typically smaller than fifty micrometres. The transverse coherence length is a few micrometres^26^, and the speckle can be generated using fine abrasive paper with smaller average grain size. A high resolution X-ray detector is usually required to resolve such a speckle pattern, but the inherently low intensity of X-rays generated by a microfocus source means that it would take a much longer period to acquire each image^26^. A higher X-ray dose and smaller field of view limit the phase-contrast based speckle technique for widespread applications using a lab-based X-ray source. Instead of using phase-based speckle^26^,^27^, we propose the use of abrasive paper with a large grain size to generate absorption-based “speckle” to overcome these issues. Technically, the features are a random absorption contrast pattern, and not true “speckle”. For a lab-based X-ray source with smaller coherence length, the effect from the partial coherence can be neglected. Therefore, a random intensity pattern is produced via pure absorption contrast from an arbitrary distribution of abrasive paper grains. In addition to the less stringent requirements for transverse coherence, absorption-based “speckle” is also less demanding on longitudinal coherence. Sufficient speckle visibility (shown in Fig. 1b) can be achieved with a polychromatic X-ray source. Since the random pattern is hundreds of micrometres in size, a high efficiency, flat panel X-ray detector (pixel size from 50–200 μm) is able to resolve the large ‘speckle’ pattern thanks to the geometric magnification of the microfocus X-ray source. Data acquisition time is dramatically reduced to less than one second per image, and the delivered dose minimized accordingly. This potentially enables the technique to be transferred to clinical applications which often require a low X-ray dose.

Having created a speckle pattern, the next step is to retrieve X-ray absorption, differential, phase and dark-field images simultaneously by scanning the abrasive paper in only one direction. The inherent 2D and random nature of a speckle pattern allows each individual speckle to be used as a wavefront marker. As shown in Fig. 1, a series of speckle images are captured by an X-ray detector without the sample present as abrasive paper is moved perpendicular to the X-ray beam using a motorized linear translation stage. The procedure is then repeated with the sample inserted downstream of the abrasive paper. To aid clarity, we discuss only the vertical scan case. When the abrasive paper is scanned along the vertical direction 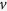, the intensity signal oscillates as a function of 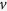 at pixel 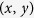. As demonstrated in Fig. 2, 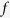 and 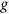
represent the speckle pattern at pixel 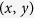 before and after adding the sample. Few nearby pixels along the horizontal direction (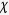) are selected in order to track the speckle displacement in two transverse directions. The correlation coefficient map γ is then generated from the two speckle arrays:


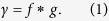


Here cross-correlation is represented by the star symbol. A sub-pixel registration algorithm is employed to precisely locate the maximum of the cross-correlation coefficient 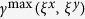
28. The coordinate 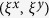 is related to the speckle displacement induced by the sample, while the magnitude 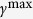 indicates the speckle distortion, which provides the scattering angle distribution caused by the sample^24^,^29^. Following a basic geometric relationship, the wavefront gradient 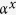, 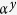 along the horizontal and vertical directions can be directly calculated from the speckle displacement


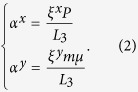


where *μ* and *P* are the scanning step size and detector’s pixel size respectively. Here, 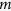 is the geometrical magnification 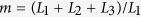. Distances between the source, abrasive paper, sample and detector are represented by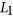, 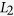
and 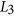. The first derivative of the sample’s phase shift 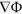 can be directly calculated from the wavefront gradients, and the phase shift 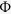 induced by the sample can then be reconstructed from the two transverse phase gradients^30^.

The dark-field signal D, related to internal structure inhomogeneity, or electron density variations, can be calculated from the maximum correlation coefficient 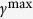. The absorption image, A, is obtained from the ratio of the sum of the two arrays 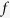 and 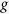 collected after compensating the speckle displacement for each pixel. Therefore, two orthogonal wavefront gradients, phase image, dark-field image and absorption image can simultaneously be acquired whilst translating the abrasive paper in only one direction. Compared to traditional 2D raster scanning, this novel approach dramatically reduces the number of scans required, thereby reducing the X-ray dose delivered to the sample. Although phase shift can be calculated by performing 1D integration, artefacts will be produced along the direction of integration due to propagation of statistical errors^30^. Such issues can be removed by integration using two orthogonal gradients, which are generated simultaneously by the proposed speckle-based method.

## Results

**Figure 2:**
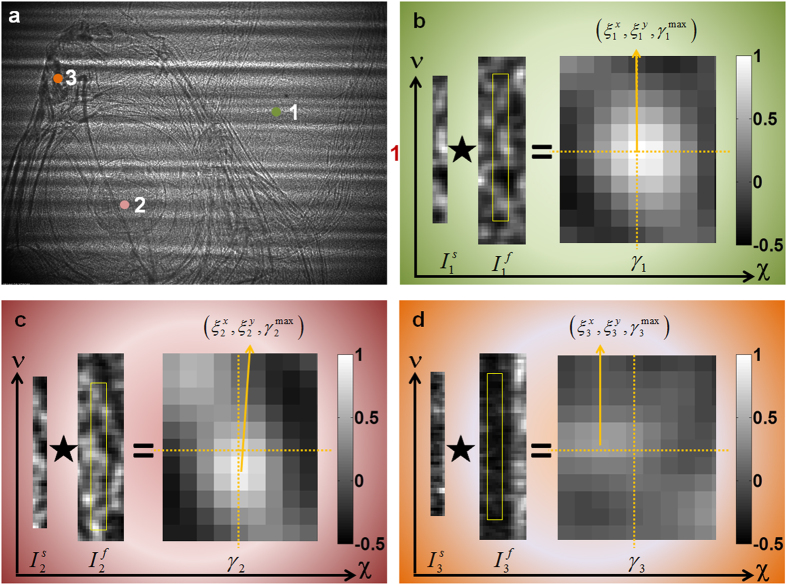
Illustration of the basic principle of the speckle-based X-ray imaging technique. Speckle image of a fish head. Speckle patterns at three representative positions (1 = empty space, 2 = eye, 3 = nostril) are indicated in (**a**). Corresponding correlation coefficient maps for positions 1, 2 and 3 are shown in (**b**), (**c**), and (**d**) respectively. Horizontal (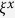) and vertical (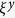) displacements and maximum correlation coefficients can simultaneously be extracted from the correlation coefficient map, and provide valuable information about localised scattering.

The principle of our technique was first validated with synchrotron X-rays at Diamond Light Source’s B16 Test beamline^31^. Figure 2 shows one speckle image from the first stack of images applied to a small fish sample. Speckle images from the stack before and after adding the sample for position 1 (empty space), 2 (eye) and 3 (nostril) are displayed in Fig. 2b–d. A cross-correlation algorithm was employed to analyse two speckle images with (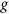) and without (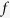) the sample present. Distinct changes due to the presence of the sample can clearly be observed in the correlation coefficient maps (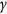). Orthogonal displacements and dark-field information can simultaneously be derived from the correlation coefficient map. Speckle patterns should not change for empty space and, as expected, Fig. 2b shows that for this case the speckle displacement for the horizontal 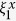 and vertical 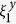
directions is 0, and the maximum of the cross-correlation coefficient 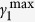 is approximately equal to 1. However, for positions 2 and 3, significant vertical and horizontal displacements of the speckles are observed in the corresponding correlation coefficient maps. This indicates that the phase gradient is quite different for these two positions. Moreover, the maximum correlation coefficient for position 3 is much lower than for position 2, indicating severe distortion of the speckle pattern at position 3. This result agrees with the expectation of stronger scattering from the fish’s nostril compared to the eye region.

**Figure 3:**
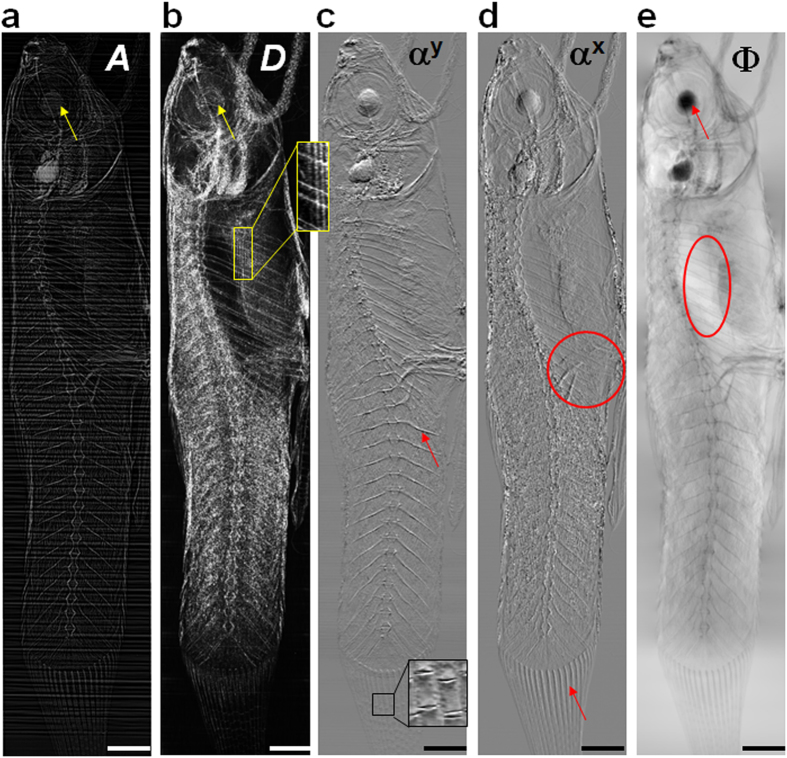
(**a**) Absorption, (**b**) dark-field, (**c**) vertical and (**d**) horizontal differential phase gradient, and (**e**) phase contrast images of a fish. Each technique is shown to identify different types of structures within the sample. All five types of image can be simultaneously acquired from a single dataset using our new 1D scanning technique. The scale bar at the bottom right corner of each image is 1 mm long.

Following the above algorithm, pixel-wise analysis was performed for two stacks of speckle images to simultaneously produce maximum correlation coefficient and vertical and horizontal displacement maps. Absorption, dark-field, two wavefront gradients, and phase images for the fish are shown in Fig. 3. Striking differences can be observed in these images. As expected, the conventional absorption image (Fig. 3a) provides substantial contrast between soft tissue and fish bones. However, image quality has been deteriorated by irregular stripes caused by the monochromator. Such stripes can be treated as low frequency speckle patterns in the proposed technique, and the derived dark-field, wavefront gradient and phase images are unaffected. As anticipated, strong scattering is observed from the nostrils, gills, and bones in the dark-field image (Fig. 3b). Internal structure in the pectoral fin (enclosed by a rectangle) is clearly seen in the dark-field image. It should be mentioned that the retrieved dark-field signals can be treated as the superposition of the horizontal and vertical directional dark-field signals. In order to study the directional scattering information, it is possible to retrieved directional dark-field image from same data set [see Supplemental Material]. This highlights that the dark-field image can reveal features caused by scattering from sub-pixel sized structures that are inaccessible from the corresponding absorption image.

As shown in Fig. 3c, horizontal fish bones are clearly visible in the vertical wavefront gradient image (Fig. 3c), whilst they are hardly noticeable in the horizontal gradient (Fig. 3d). Moreover, the connecting part between the pelvic fin and trunk (enclosed by a circle in Fig. 3d) can be clearly seen in Fig. 3c, but is barely observable in the horizontal gradient image (Fig. 3d). Dramatic differences in the fish eye and caudal fin (enclosed by a square) can also be observed in the horizontal and vertical gradient images. Therefore, we clearly demonstrate the benefit of using directional differential contrast images to investigate anisotropic sample features, especially as no extra dose is delivered to the sample as both images are extracted from a single data set using our new technique. By calculating the standard deviation of the wavefront gradient in empty space (such as the area surrounding position 1 in Fig. 2a)^22^,^23^, the angular sensitivity along both directions is found to be ~20 nrad for our experimental setup, since the step size is equal to the effective pixel size. Such angular sensitivity is also consistent with theoretical evaluation^25^, and can be further improved by scanning the abrasive paper along the particular direction of interest using a smaller step size. The phase image induced by the fish is reconstructed from the transverse phase gradient images. Unlike the conventional absorption image (Fig. 3a), soft tissues and bones can clearly be observed in the phase image (Fig. 3e). As expected, improved phase contrast is induced by soft tissue regions including the fish eye and stomach (enclosed by an ellipse), while these parts are barely visible in the corresponding dark-field image. This simple study illustrates that phase contrast and dark-field contrast images can often provide different, but complementary, information. To fully resolve high and low atomic number features within a complicated sample, it is therefore highly desirable to obtain a complete set of complementary images, which our technique can simultaneously provide.

**Figure 4:**
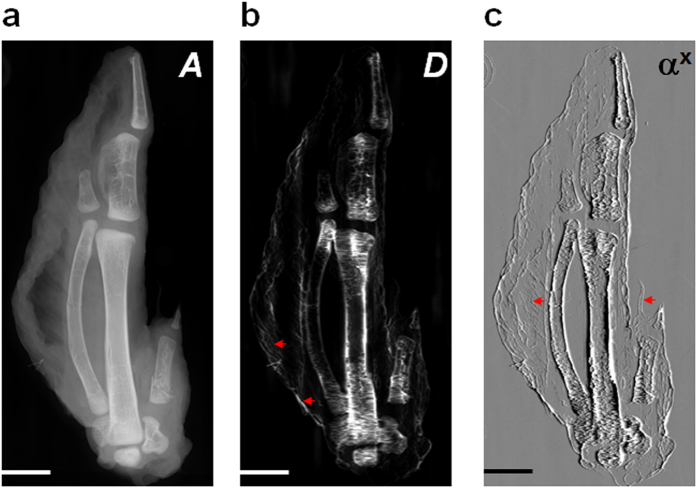
(**a**) Absorption, (**b**) dark-field and (**c**) horizontal differential phase gradient images of the tip of a chicken wing. The scale bar in the bottom left corner is 10 mm long.

To demonstrate the potential future application of the proposed technique for preclinical studies, we also investigated a biological sample using a microfocus X-ray source. As discussed above, the microfocus source provides a sufficiently small source such that the magnified absorption-based ‘speckle’ can be resolved using a flat panel X-ray detector. Figure 4 shows preliminary results for the tip of a chicken’s wing analysed using the same algorithm described above. The dark-field image (Fig. 4b) shows enhanced contrast between soft tissue and bone compared to the absorption image (Fig. 4a). Although the intensity distributions at the edges and internal parts of the bones are similar in the absorption image, distinguishable scattering information is observed in the dark-field image. Additionally, subtle features (indicated with arrows) at the edges of the soft tissue are greatly enhanced in the dark-field image, whilst the fine, inner structures of soft tissue and bone are provided by the absorption image. Figure 4c shows that vertical features are clearly visible in the horizontal wavefront gradient image. Amazingly, a fine feather (indicated by a triangle) is evident in Fig. 4c, which is hardly distinguishable in the absorption image. Once again, we have shown that our multi-imaging technique provides richer information about the sample. Additionally, the experimental arrangement offers the adaptability to achieve either a larger field of view or higher spatial resolution by simply adjusting the distance between sample and X-ray source.

## Discussion

In summary, we have developed mathematical procedures and provided experimental verification to show that our advanced, speckle-based X-ray imaging technique can simultaneously acquire absorption, orthogonal differential phase, dark-field, and phase contrast images by scanning abrasive paper in only one direction. The five images provide complementary information about different types of structures within the sample.

Compared to existing speckle-based methods which use 2D raster scans, the data acquisition period and corresponding radiation dose are dramatically reduced for our 1D scanning technique. In addition to use with a monochromatic X-ray beam from a high brilliance synchrotron radiation source, the proposed technique is also shown to be fully compatible with polychromatic X-rays generated by a microfocus lab source. Such sources are more readily available, compact, and easily implemented for practical applications. Despite the fact that the inherent flux from a microfocus source is considerably lower than from a conventional X-ray tube, the data acquisition time can be further reduced by using a high efficiency, flat panel detector which is commonly used for clinical imaging. The experimental arrangement for the proposed technique is simple, and the requirement for mechanical stability is less stringent than for other techniques. Angular sensitivity can be further increased by improving experimental parameters and employing advanced sub-pixel registration algorithms^28^. Moreover, the proposed approach can also benefit from use of a high brilliance X-ray lab-based source, such as a liquid-metal-jet source^26^. Compared to recently developed grating-based or edge illumination techniques, the use of abrasive paper is also highly advantageous for several reasons including: extremely low cost; high X-ray transmission; large field of view; and simple alignment. Moreover, various grades of abrasive paper with different grain sizes enable the proposed technique to be applied to medical and industrial imaging using a wide range of X-ray detectors. As a consequence, these exceptional features allow the technique to overcome many of the limitations suffered by existing X-ray imaging methods. Although biological samples were used in this study, the technique can in principle be applied to other types of samples and sophisticated materials in X-ray radiography and tomography. We sincerely hope for widespread use of this technique in the near future as it could provide valuable improvements for X-ray multi-mode imaging for biomedical research and preclinical applications.

## Methods

Apart from the rigid translation due to phase shift, the speckle pattern may also become distorted because of small angle scattering from the sample. In previous work, only the phase shift has been considered to be part of the optical transfer function^25^. In this study, in addition to the phase shift, we also take into account the sample’s scattering, which is assumed to be isotropic with a Gaussian distribution^10^. In the small angle scattering approximation, the optical transfer function can be expressed as^32^


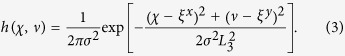


Here, 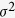
is the effective second moment of the total scattering angle distribution^33^. 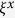 and 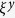 is the displacement between two speckle patterns 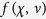 and 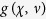 along horizontal and vertical direction, respectively. The cross-correlation between the signal recorded in the absence 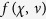 or presence 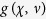 of the sample at each detector pixel 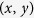 with a global translation 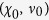 is given by^34^


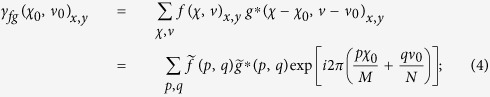


where: 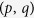 are Fourier space variables; *M* and *N* are the image dimensions; (*) denotes complex conjugation; and the operator ~ denotes the Fourier transform. With the sample present, the speckle intensity pattern 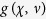 is the convolution of the pattern without the sample 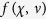 and the optical transfer function 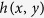. According to the convolution theorem of the Fourier transform^35^, it can be expressed as:


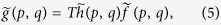


Here 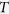 is the intensity scale factor, which depends on the transmission of the sample.

The Fourier transform of a Gaussian function 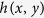 is,


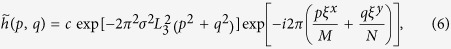


where 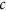 is appropriate constant. The displacement 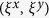 between two speckle patterns is phase shift term in Fourier space based on the shifting theorem of the Fourier transform^35^.

Inserting Eq. (5) and (6), Eq. (4) can be rewritten as


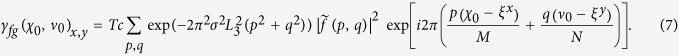


The cross-correlation is maximum at 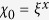 and 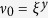, and the displacement of the speckle pattern induced by the sample can be obtained accordingly. The wavefront gradient can then be calculated from Eq. (2). Therefore, the maximum of the cross-correlation map is given by


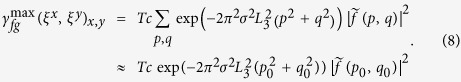


where frequencies 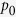 and 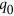 correspond to the maximum of the power spectrum 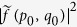, and are related to the average speckle size 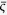 by  
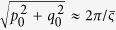
. Using normalized cross-correlation, the scaling factor 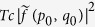 will be unity and 
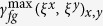
 thus can be simplified as 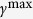 with


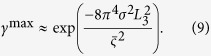


The dark-field signal *D* can be defined as the second moment of the scattering angle distribution^32^:


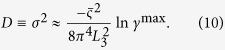


The previous approach for directional dark-field imaging provided only qualitative scattering information^29^. In contrast, Eq. (10) quantitatively describes the relationship between *D* and 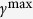. Therefore, the dark-field signal fulfils the line integral condition^33^, and can be extended for three-dimensional (3D) X-ray dark-field computed tomography using conventional reconstruction methods.

Once speckle displacement 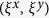 has been retrieved, the new speckle array can be generated by compensating the speckle displacement with 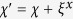 and 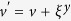. The absorption image *A*, can be calculated from the transmission *T*, which is defined as the ratio of the mean (^–^) of two arrays 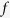 and 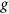
in each pixel:


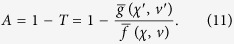


The synchrotron light experiment was performed at Diamond Light Source’s B16 Test beamline^31^. A monochromatic beam of X-rays of energy 20 keV was selected from the broadband source using a double multilayer monochromator (ΔE/E ≈ 10^−2^). Abrasive paper with an average particle size of 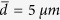
was mounted on a motorized linear stage located at 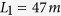 from the X-ray source. A small fish (*Poecilia wingei*) was placed on an independent motorized linear translation stage 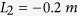 upstream of the abrasive paper. Abrasive paper and detector were set to the maximum mechanical separation of 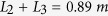 to improve angular sensitivity. The X-ray camera used to record speckle images was based on a PCO 4000 CCD detector and used a Ce-doped YAG scintillator. A 10× microscope objective was equipped with an effective pixel size of 1.8 μm × 1.8 μm after 2 × 2 binning. A stack of 60 images was collected without the sample present in the X-ray beam by scanning the abrasive paper vertically with a step size of μ = 1.8 μm. Data acquisition for each speckle image took 0.4 s. In this study, the speckle visibility varied from 0.20 to 0.25. Since the effective field of view of the X-ray camera is only 3 mm (horizontal) × 1.5 mm (vertical), 15 stacks of images were collected for each position by translating the fish vertically. Subsequently, absorption, dark-field, and horizontal and vertical differential phase images were extracted with pixel-wise analysis using a cross-correlation algorithm for each position. An advanced, phase correlation method was employed to stitch 15 images together for the four image modes^36^. The phase contrast image was then reconstructed by integrating the two stitched orthogonal differential phase images, to produce the results presented in Fig. 3.

The proposed technique was then extended to use a Nikon XSTH 225 microfocus X-ray source. The X-ray tube operates at 50 kV peak voltage and 450 μA current. To increase absorption contrast for the random pattern, a sheet of silicon carbide abrasive paper with large particle size (FEPA Grit P80 with 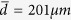) was placed close to the source (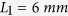). A molybdenum reflection target was used to generate X-rays with a focal spot size of 20 μm × 20 μm, which is sufficiently small to produce absorption ‘speckle’ images from the large silicon carbide particles. The transverse coherence length is less than one micrometer in this case, therefore, the absorption contrast plays more important role compared to the effect from the partial coherence. The high dynamic range of the flat panel X-ray detector (Perkin Elmer 1620) with 2000 × 2000 pixels (pixel size: 200 μm × 200 μm) was employed to record the magnified absorption-based ‘speckle’. The resolution of the imaging system is mostly limited by the source size due to higher geometrical magnification 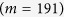. The exposure time for a single raw image was only 0.5 s. In total, 60 raw images were recorded by scanning the abrasive paper along the vertical direction with a step size of μ = 10 μm, which is the minimum step size for the existing motorized stage. Here we would like to emphasize that the total data acquisition time (t = 30 s) for the proposed technique is comparable to its counterpart (t = 40 s) using a Talbot-Lau grating interferometer^14^, but the power (22.5 W) of the X-ray microfocus source is much lower than from a conventional X-ray tube (1200 W). In addition to the simpler experimental setup, a lower X-ray dose is induced to the sample for the proposed technique. The tip of chicken wing was placed in a polystyrene box, and the sample to abrasive paper distance was set to 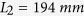 such that the whole sample was visible in the field of view. The total distance from the source to detector was 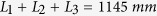. Even though the vertical scan mode was used in this experiment, interestingly the horizontal angular sensitivity is nearly 10 times better than the vertical direction due to the higher geometrical magnification. A separate horizontal scan can be performed along horizontal direction in order to increase the angular sensitivity along vertical direction [see Supplemental Material]. Since the angular sensitivity is relative low due to some constrains of existing experimental setup, such as high geometrical magnification, coarse step size etc, the sensitivity can be further improved by optimizing the experimental parameters, using high precision linear stage according to Eq. (2).

## Additional Information

**How to cite this article**: Wang, H. *et al.* From synchrotron radiation to lab source: advanced speckle-based X-ray imaging using abrasive paper. *Sci. Rep.* **6**, 20476; doi: 10.1038/srep20476 (2016).

## Supplementary Material

Supplementary Information

## Figures and Tables

**Figure 1:**
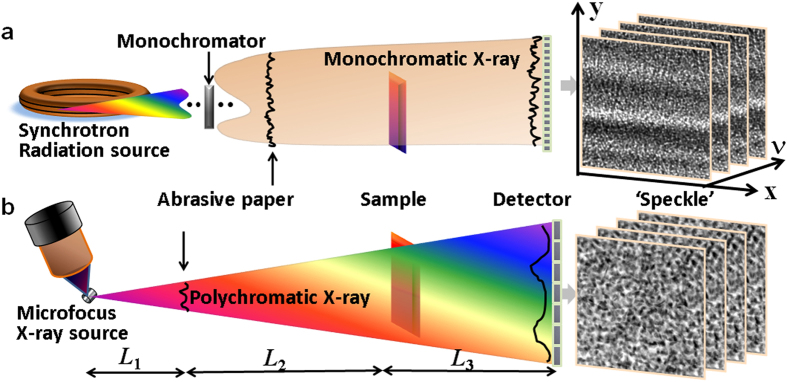
Schematic representation of the experiment setup for (a) synchrotron radiation source and (b) microfocus X-ray source. (**a**) A stack of phase-based speckle images is resolved using a high resolution X-ray detector by scanning abrasive paper transversely to the monochromatic X-ray beam from a synchrotron radiation source. (**b**) The corresponding absorption-based “speckle” images recorded by flat panel detector are obtained by placing large grain, abrasive paper close to the polychromatic X-ray microfocus source.
