# Supplementary material for: From synchrotron radiation to lab source: advanced speckle-based X-ray imaging using abrasive paper
Source: Sci Rep. 2016 Feb 5;6:20476. doi: 10.1038/srep20476 (PMC4742822; doi:10.1038/srep20476)
Supplement: Supplementary Information [file srep20476-s1.doc]

**SUPPLEMENTARY INFORMATION**

**From synchrotron radiation to lab source: advanced speckle-based X-ray imaging using abrasive paper**

Hongchang Wang*, Yogesh Kashyap and Kawal Sawhney

# Comparison of dark-field and directional dark-field images for the specimen shown in Fig. 3 of the manuscript

In the proposed method, the speckle displacement is tracked along horizontal and vertical directions simultaneously; hence the retrieved dark-field image from effective cross-correlation decrement is due to speckle distortion from orthogonal directions. As shown in Fig.1S (a), the retrieved dark-field signals can be treated as the superposition of the orthogonal directional dark-field signals. As highlighted in the enclosed rectangle region of the dark-field image (Fig. 1S (c)), both the horizontal and vertical directional dark-field signals from fish caudal fin are retrieved from single vertical scan. In addition, the directional dark-field signal can be retrieved from the same data set by considering the correlation decrement along a single direction[^1^](#_ENREF_1). As shown in Fig.1S (b), the retrieved vertical dark-field signal is dominated by horizontal features. In contrast to the dark-field image in Fig.1S (a), the horizontal fish bone can be clearly distinguished from the soft tissues in the retrieved vertical dark-field image. To look more closely in Fig. 1S (d), only the horizontal features (thin arrow) of the fish caudal fin are visible, while the vertical scattering features (thick arrow) disappeared. It should be mentioned that both dark-field and directional dark-field images are extracted from a single data set, and it will be valuable for the study of strongly ordered systems.


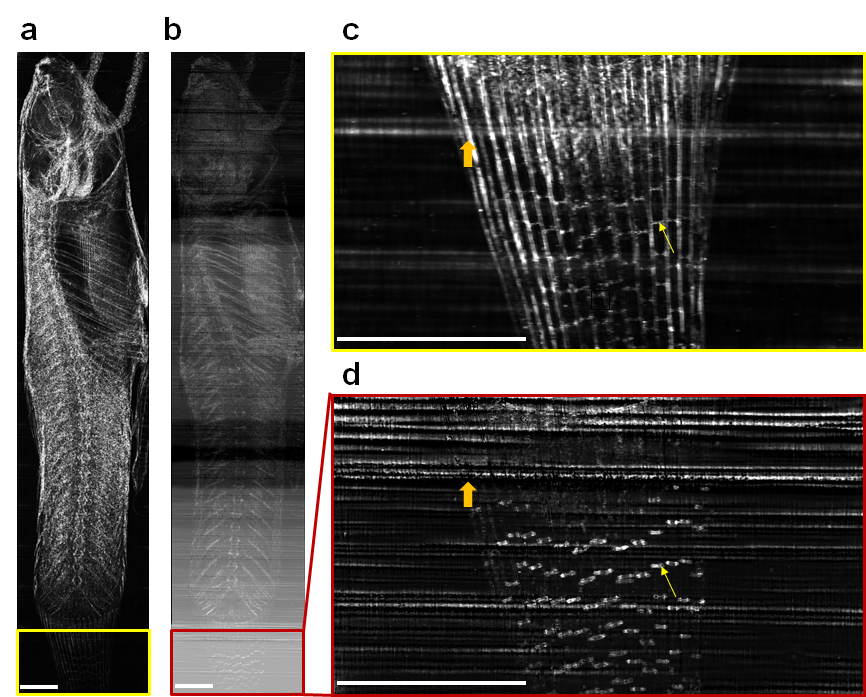


FIG. 1S.  **(a) dark-field image and (b) vertical dark-field image. (c) and (d) are magnified and contrast-optimized parts (enclosed by the rectangle regions) of image (a) and (b).** The scale bar at the bottom left corner of each image is 1mm long.

# Multiple images from lab-based source

In order to demonstrate the feasibility of the proposed technique to retrieve multiple images with a micro-focus X-ray source, we present a study of a fish (sprat). The experimental setup is same with the one described in Fig. 4 of the manuscript. Even though the vertical scan mode was used in this experiment, interestingly the horizontal angular sensitivity is nearly 10 times better than the vertical direction due to the higher geometrical magnification. Therefore, an additional horizontal scan was performed along horizontal direction in order to improve the angular sensitivity along vertical direction, and the phase shift image was then reconstructed from the two transverse phase gradients[^2^](#_ENREF_2). The retrieved absorption, dark-field, vertical and horizontal differential phase gradient, and phase contrast images are shown in Fig. S2. As described in the manuscript, these images provide complementary information to each other. It should be noted that larger field of view can be achieved with lab-based source compared to the synchrotron radiation source. However, we may point out that the sensitivity of the phase gradient from lab-based X-ray source is lower compared to the one from synchrotron radiation source. Since the phase image contrast is relative low due to some constrains of existing experimental setup, such as high geometrical magnification, coarse step size etc., the sensitivity can be further improved by optimizing the experimental parameters and using a high precision linear stage. Moreover, the proposed approach can also benefit from use of a high brilliance X-ray lab-based source, such as a liquid-metal-jet source[^3^](#_ENREF_3).


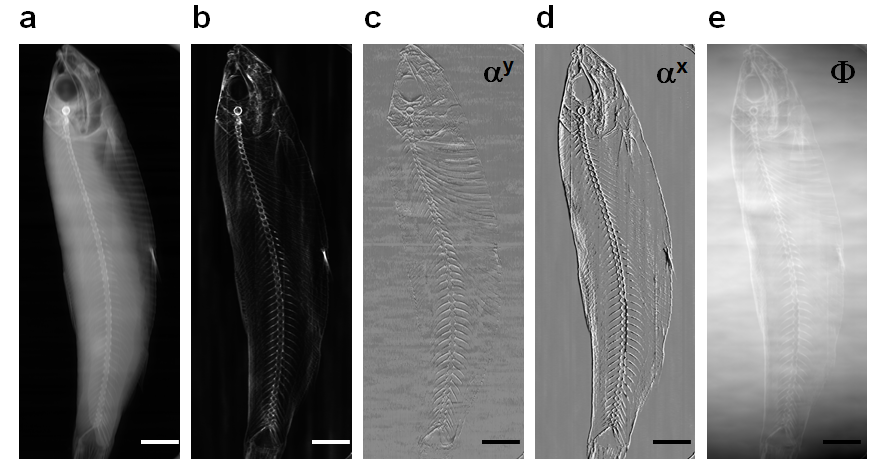


FIG. 2S. **| (a) Absorption, (b) dark-field, (c) vertical and (d) horizontal differential phase gradient, and (e) phase contrast images of a sprat fish.** Each technique is shown to identify different types of structures within the sample. The scale bar at the bottom right corner of each image is 10mm long.

-------------------------

*hongchang.wang@diamond.ac.uk

1 Wang, H., Kashyap, Y. & Sawhney, K. Hard-X-Ray Directional Dark-Field Imaging Using the Speckle Scanning Technique. Phys. Rev. Lett. **114**, 103901 (2015).

2 Kottler, C., David, C., Pfeiffer, F. & Bunk, O. A two-directional approach for gratingbased differential phase contrastimaging using hard x-rays. Opt. Express **15**, 1175-1181 (2007).

3 Zanette, I. et al. Speckle-Based X-Ray Phase-Contrast and Dark-Field Imaging with a Laboratory Source. Phys. Rev. Lett. **112**, 253903 (2014).
